# Different Sensitivity of Control and MICU1- and MICU2-Ablated *Trypanosoma cruzi* Mitochondrial Calcium Uniporter Complex to Ruthenium-Based Inhibitors

**DOI:** 10.3390/ijms21239316

**Published:** 2020-12-07

**Authors:** Mayara S. Bertolini, Roberto Docampo

**Affiliations:** Center for Tropical and Emerging Global Diseases and Department of Cellular Biology, University of Georgia, Athens, GA 30606, USA; maybertolini@uga.edu

**Keywords:** calcium, mitochondrial calcium uniporter, ruthenium red, *Trypanosoma cruzi*

## Abstract

The mitochondrial Ca^2+^ uptake in trypanosomatids shares biochemical characteristics with that of animals. However, the composition of the mitochondrial Ca^2+^ uniporter complex (MCUC) in these parasites is quite peculiar, suggesting lineage-specific adaptations. In this work, we compared the inhibitory activity of ruthenium red (RuRed) and Ru360, the most commonly used MCUC inhibitors, with that of the recently described inhibitor Ru265, on *Trypanosoma cruzi*, the agent of Chagas disease. Ru265 was more potent than Ru360 and RuRed in inhibiting mitochondrial Ca^2+^ transport in permeabilized cells. When dose-response effects were investigated, an increase in sensitivity for Ru360 and Ru265 was observed in *TcMICU1*-KO and *TcMICU2*-KO cells as compared with control cells. In the presence of RuRed, a significant increase in sensitivity was observed only in *TcMICU2*-KO cells. However, application of Ru265 to intact cells did not affect growth and respiration of epimastigotes, mitochondrial Ca^2+^ uptake in Rhod-2-labeled intact cells, or attachment to host cells and infection by trypomastigotes, suggesting a low permeability for this compound in trypanosomes.

## 1. Introduction

The mitochondria have a fundamental role in intracellular Ca^2+^ homeostasis, buffering cytosolic Ca^2+^ increases arising from both influx from the extracellular space and release from intracellular organelles. Ca^2+^ is taken up by a channel known as mitochondrial Ca^2+^ uniporter (MCU), and once inside, it stimulates several dehydrogenases [1,2] and the ATP synthase [3,4] to generate ATP. The MCU was found almost sixty years ago in kidney mitochondria [5,6], but its molecular identity was discovered more recently [7,8]. The discovery that *Trypanosoma cruzi*, the agent of Chagas disease, possesses a mitochondrial Ca^2+^ uptake mechanism with similar characteristics to those of animal mitochondria [9,10], combined with the finding of the absence of the channel in *Saccharomyces cerevisiae* [11] and the availability of the genomes of these species, was important for the molecular identification, first of a modulator of the channel, mitochondrial Ca^2+^ uptake 1 (MICU1) [12] and then of the pore subunit of the channel or MCU [7,8]. After this discovery several accessory proteins were found, like MCU regulator 1 (MCUR1) [13], MICU2 and MICU3 [14], MCUb [15], and essential MCU regulator (EMRE) [16], comprising an MCU complex (uniplex or holocomplex). *T. cruzi* MCU complex, however, does not possess the MCUR1, MICU3 or EMRE components of the animal uniporter and, in contrast, possesses four Ca^2+^-conducting subunits: MCU, MCUb, MCUc, and MCUd [17]. Two of these subunits, MCUc and MCUd, are only present in trypanosomatids [17,18]. These four subunits form hetero-oligomers, probably hetero-hexamers [18]. Trypanosomatid MCU complexes also differ from those of animals in that the MCUb subunit is a Ca^2+^-conducting subunit and does not have a dominant-negative activity on the MCU complex [18,19]. In addition, trypanosomes MICU1 and MICU2 do not form dimers linked by disulfide bonds and do not have individual gatekeeping activity preventing Ca^2+^ influx through the uniporter at low Ca^2+^ concentrations [20], as the animal proteins have [21,22,23]. All these characteristics are suggestive of the parallel evolution of the complex in trypanosomes [24], which belong to the Discoba supergroup of eukaryotes, and animal cells, which belong to the Opisthokonta supergroup.

Early work demonstrated the inhibition of mitochondrial Ca^2+^ uptake in *T. cruzi* by ruthenium red (RuRed) [10]. However, Ru360, which is the most commonly used MCU inhibitor in animal cells, was never tested on trypanosomes. Ru360 is a binuclear oxo-bridged ruthenium ammine complex that has high absorbance at 360 nm [25]. Site-directed mutagenesis of the S259 residue near the pore motif (DIME) of human MCU to alanine resulted in partial resistance to Ru360 inhibition, suggesting that this amino acid is important for the inhibition [8]. Interestingly, all the *T. cruzi* monomers of the MCU complex lack this serine residue [17]. Other work was consistent with the binding of RuRed/Ru360 with the aspartate(D)-ring of MCU’s selectivity filter [26,27], which is within the second transmembrane domain of the MCU monomer [28] and is solvent-exposed in contrast to the glutamate(E)-ring located deeper in the pore. It was also found that MICU1 suppresses the inhibition of MCU by RuRed/Ru360, which binds to the DIME motif of MCU through a DIME-interacting domain (DID) [29]. Given that the *T. cruzi* mitochondrial uniporter differs extensively from the animal uniporter, we explored whether ruthenium derivatives are able to inhibit Ca^2+^ transport in *T. cruzi* and whether MICU1 or MICU2 suppresses MCU inhibition by these compounds.

In this work, we report the inhibitory activity of RuRed as compared to Ru360 and the recently described cell-permeable inhibitor Ru265 [30] on mitochondrial Ca^2+^ uptake in *T. cruzi*. We show that Ru265 is the most effective inhibitor and that this inhibition is increased in *TcMICU1*- and *TcMICU2*-knockout cells. The use of Ru265 in intact cells did not result in phenotypic changes suggesting low permeability of this compound in trypanosomes.

## 2. Results

### 2.1. Inhibition of Mitochondrial Ca^2+^ Uptake by Ruthenium-Based Compounds

**Figure 1:**
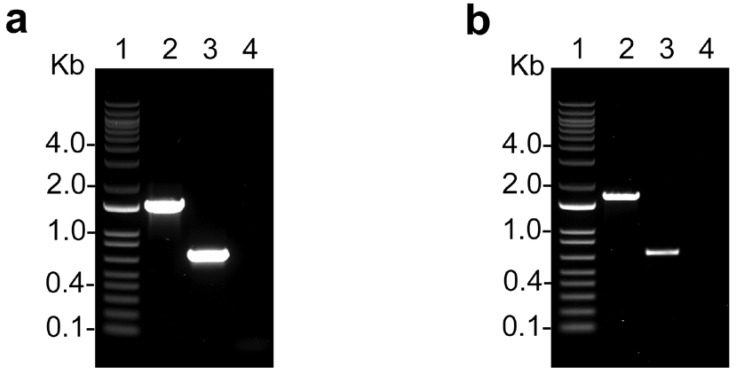
PCR confirmation of *TcMICU1*-KO and *TcMICU2*-KO. (**a**) *TcMICU1*-KO mutant was generated by CRISPR/Cas9-induced homologous recombination. A double-stranded gDNA break was produced by Cas9 at nt +70 of the *TcMICU1* ORF (1221 bp). DNA was repaired with a blasticidin-S deaminase (*Bsd*) cassette containing 100-bp homologous regions from *TcMICU1* 5′ and 3′ untranslated regions (UTRs). The gene replacement was verified by PCR. The intact locus generates a PCR product of 1544 bp, while the disrupted locus generates a fragment of 722 bp. (**b**) *TcMICU2*-KO mutant was generated by CRISPR/Cas9-induced homologous recombination. A double-stranded gDNA break was produced by Cas9 at nt +84 of the *TcMICU2* ORF (1407 bp). DNA was repaired with a blasticidin-S deaminase (*Bsd*) cassette containing 100-bp homologous regions from *TcMICU2* 5′ and 3′ untranslated regions (UTRs). The gene replacement was verified by PCR. The intact locus generates a PCR product of 1701 bp, while the disrupted locus generates a fragment of 693 bp. Lanes: 1, 1-kb ladder; 2, wild type; 3, *TcMICU1*-KO (**a**) or *TcMICU2*-KO (**b**); 4, PCR negative controls.

We first tested the effect of different concentrations of inhibitors on Ca^2+^ uptake, as measured by the change in fluorescence of calcium green 5-N, by digitonin-permeabilized control and *TcMICU1*-KO and *TcMICU2*-KO epimastigotes. These KO cells were obtained by the CRISPR/Cas9 method in previous work [20], and we used cells transfected with a scrambled sgRNA as control. Figure 1 shows the PCR confirmation of the knockouts.

**Figure 2:**
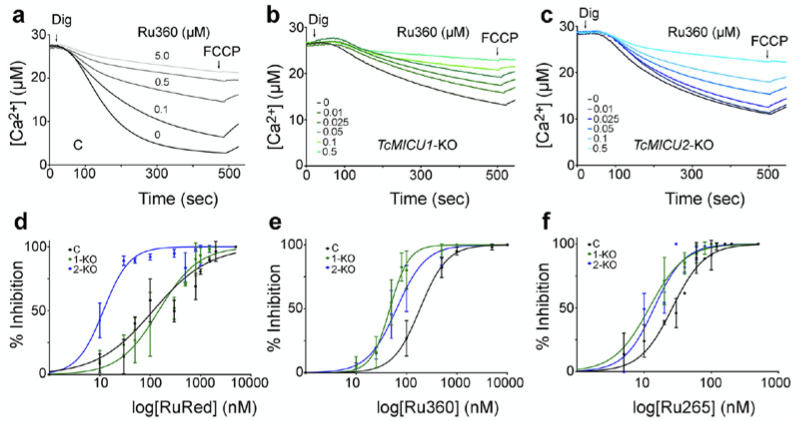
Inhibition of mitochondrial Ca^2+^ uptake by ruthenium-based compounds. Representative traces of Ca^2+^ uptake by digitonin-permeabilized epimastigotes in control (**a**), *TcMICU1*-KO (**b**), and *TcMICU2*-KO (**c**) epimastigotes in the presence of different Ru360 concentrations. C, control cells; *TcMICU1*-KO cells; and *TcMICU2*-KO cells. The reaction was started after adding 50 μM digitonin (Dig) in the presence of ~30 μM CaCl_2_, 5 mM succinate, and 0.5 μM calcium green-5N, in the presence or absence of Ru360. Where indicated, 4 μM FCCP was added. (**d**–**f**) Dose–response inhibition of the initial rate of mitochondrial Ca^2+^ uptake in control (C), *TcMICU1*-KO (1-KO), and *TcMICU2*-KO (2-KO) cells treated with RuRed (**d**), Ru360 (**e**) or Ru265 (**f**). A sigmoidal fit is displayed for each.

As is shown in Figure 2a, extramitochondrial Ca^2+^ was sequestered in the mitochondria of control cells and released by the uncoupler FCCP. In agreement with a previous report [20], both *TcMICU1*-KO and *TcMICU2*-KO epimastigotes showed a reduced capacity for mitochondrial Ca^2+^ uptake (Figure 2b,c). This activity was inhibited by Ru360, added prior to the delivery of 30 µM Ca^2+^ in the presence of succinate as an energy source, in a dose-dependent manner, and with an IC_50_ of 185.9 ± 15.2 nM. As shown in Figure 2e, a sigmoidal fit is displayed for this inhibition. RuRed (Figure 2d) and Ru265 (Figure 2f) also inhibited mitochondrial Ca^2+^ uptake in a dose-dependent mode with IC_50_s of 117 ± 16.9 nM and 26.3 ± 2.0 nM, respectively.

When dose–response effects were measured in *TcMICU1*-KO and *TcMICU2*-KO cells we noticed that these cells were, in general, more sensitive to the inhibitors. For example, IC_50_s values for Ru360 in *TcMICU1*-KO and *TcMICU2*-KO cells were 46.7 ± 2.7 nM (*p* < 0.0001) and 63.6 ± 6.9 nM (*p* < 0.0002), respectively (Figure 2e), and the IC_50_ values for Ru265 were 12.1 ± 2.3 nM (*p* < 0.003) and 14.1 ± 1.3 nM (*p* < 0.007), respectively (Figure 2f). In contrast, in the presence of RuRed a significant increase in sensitivity was observed only in *TcMICU2*-KO cells with an IC_50_ of 11.9 ± 1.4 nM (*p* < 0.005), while for *TcMICU1*-KO cells the IC_50_ was similar to that of control cells (148 ± 21.7 nM) (*p* < 0.353, n.s.) (Figure 2d).

To determine whether the defect in mitochondrial Ca^2+^ uptake in the presence of inhibitors was not secondary to mitochondrial membrane depolarization, we measured the mitochondrial membrane potential (Δψ_m_) of digitonin-permeabilized epimastigotes using safranine O in the presence of succinate as mitochondrial substrate.

**Figure 3:**
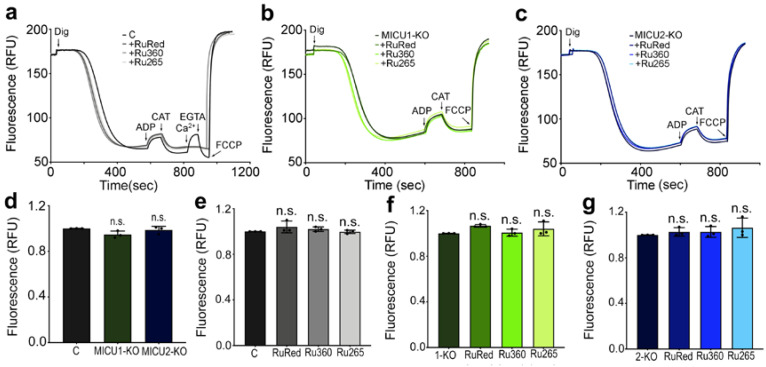
Effect of inhibitors on the mitochondrial membrane potential of epimastigotes. Changes in mitochondrial membrane potential (Δψ_m_) of digitonin-permeabilized epimastigotes as detected by changes in safranine O fluorescence in control (**a**), *TcMICU1*-KO (**b**), and *TcMICU2*-KO (**c**) epimastigotes in the absence or presence of inhibitors. Cells (5 × 10^7^) were added to the reaction buffer (2 mL) containing 0.2% BSA, 5 mM succinate, 50 μM EGTA and 5 μM safranine O. The reaction was started with 50 μM digitonin (DIG) in the presence or absence of the inhibitor, and 250 μM ADP, 1.5 μM carboxyatractyloside (CAT), 50 μM CaCl_2_ (Ca^2+^), 200 μM EGTA, and 4 μM FCCP were added when indicated. A decrease in fluorescence after permeabilization with digitonin indicated the accumulation of the dye in energized mitochondria. The addition of ADP produced a small dissipation of membrane potential, indicating ADP phosphorylation. Δψ_m_ returned to its initial level after the addition of the adenine nucleotide translocator inhibitor CAT. The addition of FCCP collapsed the membrane potential. (**d**) Changes in safranine O fluorescence after digitonin addition (between 30 and 60 s) in control (C), *TcMICU1*-KO and *TcMICU2*-KO epimastigotes. Values are means ± SD (*n* = 3). n.s., no significant differences by one-way ANOVA with Dunnett’s multiple comparisons test. Changes in safranine O fluorescence in control (C) (**e**), *TcMICU1*-KO (1-KO) (**f**), and *TcMICU2*-KO (2-KO) (**g**) epimastigotes after addition of ADP from three experiments like that shown in panels (**a**–**c**). In the panels, (**e**–**g**), values are means ± SD (*n* = 3). n.s., no significant differences by one-way ANOVA with Dunnett’s multiple comparisons test.

Figure 3a–c show that the addition of ADP to these preparations caused a small decrease in the Δψ_m_ that returned to its normal level when the adenine nucleotide translocator (ANT) inhibitor carboxyatractyloside was added to inhibit ADP/ATP exchange, while the addition of FCCP collapsed Δψ_m_. The presence of either RuRed, Ru360 or Ru265 did not significantly affect the Δψ_m_ of control, *TcMICU1*-KO or *TcMICU2*-KO (Figure 3d) cells, or their response to ADP (Figure 3e–g). Figure 3a shows that the addition of Ca^2+^ caused membrane depolarization in control cells that returned to basal levels after the addition of EGTA, but no changes were observed when ruthenium-derivatives were present.

### 2.2. Effects of Ru265 on Trypanosoma cruzi

Knockout of different subunits of *T. cruzi* MCU complex results in phenotypic changes that include delayed growth, alterations in their respiratory rate, and defects in trypomastigote invasion and amastigote replication [17,19]. Because Ru265 is apparently more cell-permeable than other ruthenium derivatives [30], we tested whether it affects epimastigote growth, and respiration, and trypomastigote attachment to host cells, which is an energy-requiring process required for host cell invasion [31].

**Figure 4:**
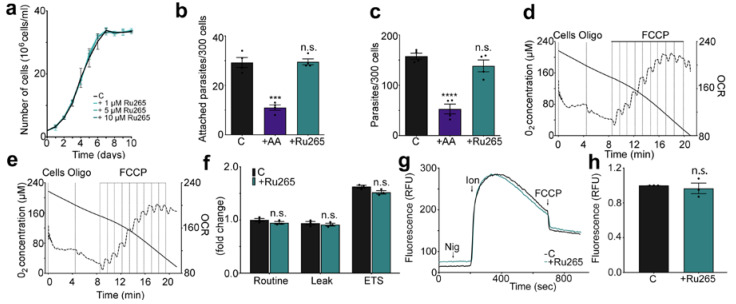
Effect of Ru265 on *Trypanosoma cruzi*. (**a**) Effect of Ru265 on epimastigote growth. Growth of control epimastigotes in LIT medium in the presence of 1 μM, 5 μM, and 10 μM Ru265. (**b**) Effect of Ru265 on trypomastigote attachment. Trypomastigotes were pretreated for 30 min with 1 μM antimycin A (AA) or 10 μM Ru265 and added to glutaraldehyde-fixed Vero cells. After 1 h of incubation at 37 °C, the cells were washed, and the number of attached trypomastigotes was counted. Values are means ± SD (*n* = 4). n.s., no significant differences; *** *p* < 0.001; by one-way ANOVA with Dunnett’s multiple comparisons. (**c**) Effect of Ru265 on trypomastigotes infection. Trypomastigotes were pretreated for 30 min with 1 μM antimycin A or 10 μM Ru265 and added to Vero cells. After 4 h of incubation at 37 °C, the cells were washed, and the number of intracellular parasites was counted. Values are means ± SD (*n* = 4). n.s., no significant differences; **** *p* < 0.0001; by one-way ANOVA with Dunnett’s multiple comparisons test. n.s., no significant differences by Student’s *t*-test. (**d**,**e**) Representative traces of oxygen consumption rate (OCR in Pmol/sec.mg) in control epimastigotes after preincubation without (**d**) or with (**e**) 10 μM Ru265 for 5 min. The dashed lines indicate the variation in oxygen concentration as a function of time (right axis). The solid lines represent O_2_ concentration (left axis). Where indicated, 0.5 μg/mL oligomycin (oligo) was added, followed by sequential additions of FCCP (0.2 μM each). (**f**) Routine respiration (initial oxygen flux values), leak respiration after the addition of 0.5 µg/mL of oligomycin A, and electron transfer system (ETS) capacity after the sequential additions of FCCP (0.2 μM each) were measured for each condition. Values are means ± SD (*n* = 3). n.s, no significant differences, by two-way ANOVA with Sidak’s multiple-comparison test. (**g**) Representative experiment showing mitochondrial Ca^2+^ uptake by intact epimastigotes in relative fluorescence units (RFU). Cells (5 × 10^7^) were loaded with the mitochondrial calcium indicator Rhod-2 AM and pretreated with or without 50 μM Ru265 for 30 min. Cells were incubated with 1 μM nigericin (Nig) and 1 μM ionomycin (Ion) to rapidly elevate cytosolic Ca^2+^ levels and induce mitochondrial Ca^2+^ uptake. The addition of 8 μM FCCP increased the release of mitochondrial Ca^2+^. (**h**) Changes in fluorescence (before and after ionomycin addition) from three experiments like that shown in panel (**g**). Values are means ± SD (*n* = 3), n.s., no significant differences.

We analyzed the growth rate of epimastigotes in LIT medium at different Ru265 concentrations (1 µM, 5 µM, and 10 µM). This assay revealed that Ru265 does not affect the growth rate of control parasites and is nontoxic at the concentrations tested (Figure 4a). The presence of metabolic inhibitors affects attachment and invasion of *T. cruzi* trypomastigotes to mammalian cells since these are an active process which depends on the expenditure of parasite energy [31]. Preincubation of trypomastigotes for 30 min with antimycin A, which blocks electron transport from cytochrome *b* to *c*_1_, decreased the ability of trypomastigotes to attach to glutaraldehyde-fixed cells (Figure 4b) and invade intact host cells (Figure 4c). However, the ability of trypomastigotes to attach glutaraldehyde-fixed cells and invade intact cells was not affected by a similar preincubation with 10 µM Ru265.

To measure the effect of this compound on parasite respiration, epimastigotes were preincubated in the presence (Figure 4d) or absence (Figure 4e) of Ru265. The rates of oxygen consumption by the parasites were measured after the addition of cells, oligomycin A, and finally uncoupled by sequential additions of FCCP to determine the routine respiration (initial oxygen flux values), leak respiration, and the electron transport system (ETS) capacity, respectively (Figure 4f) [32]. Uncoupled respiratory control rates, which are expressed as the ratio of the uncoupled rate (state 3u) to the rate when oligomycin is present, were 1.75 ± 0.05 and 1.70 ± 0.04 for control cells in the absence and presence of Ru265, respectively (*n* = 3). Moreover, the spare respiratory capacity, which is the ability of substrate supply and electron transport to respond to an increase in energy demand, was 93.71 ± 5.87 and 91.61 ± 10.6 for control cells in the presence or absence of Ru265, respectively, (*n* = 3). This is measured by the difference between state 3u and the basal rate [32]. Our results demonstrate that Ru265 did not affect O_2_ consumption level when we compared it with that of parasites without inhibitor (Figure 4f).

To determine if Ru265 could inhibit mitochondrial Ca^2+^ uptake in intact non-permeablized cells, epimastigotes were loaded with the mitochondrial Ca^2+^ indicator Rhod-2 AM in the presence or the absence of 50 µM Ru265. Cells were treated with the combination of nigericin and ionomycin, which is known to release Ca^2+^ from neutral and acidic compartments (acidocalcisomes) [33], and Ca^2+^ was rapidly taken up by the mitochondria (Figure 4g). Rhod-2 fluorescence increased after this treatment indicating mitochondrial Ca^2+^ uptake, and slowly decreased after reaching a peak. The addition of the uncoupler FCCP increased the rate of Ca^2+^ release indicating that Ca^2+^ was being released from the mitochondria and reached a new steady-state level. There was no significant difference in the amount of Ca^2+^ taken up by control and Ru265-treated cells in three independent experiments (Figure 4h), again suggesting the low permeability for Ru265 in trypanosomes.

In conclusion, preincubations with different concentrations of Ru265 did not affect growth, respiration, mitochondrial Ca^2+^ uptake, or host cell attachment and invasion, suggesting that it is not as permeable in trypanosomes as in mammalian cells.

## 3. Discussion

The most important findings of this work are: (1) Ru360 and Ru265 are nM inhibitors of the MCU complex of *T. cruzi* with Ru265 being the most effective, with an IC_50_ of only 26.3 ± 2.0 nM; (2) ablation of either *TcMICU1* or *TcMICU2* increased the sensitivity of TcMCU complex to Ru360 and Ru265; and (3) application of Ru265 to intact cells did not significantly affect epimastigotes growth or respiration, mitochondrial Ca^2+^ uptake in intact cells, and trypomastigote attachment and invasion of host cells, suggesting low permeability in trypanosomes.

RuRed has been used routinely to inhibit the MCU complex of trypanosomes, but this is the first time a dose–response effect has been tested with mitochondria in situ and its activity compared with that of more potent inhibitors, like Ru360 and Ru265. The IC_50_ of RuRed, Ru360 and Ru265 are in the nanomolar range. However, it is important to note that while RuRed is effective in the submicromolar range [34], Ru360 is effective with an IC_50_ between 0.2 and 2 nM [25] in mammalian mitochondria, suggesting that the TcMCU complex is less sensitive to Ru360. Although ruthenium-derivatives could have secondary effects, such as stimulation of Ca^2+^ release from the endoplasmic reticulum (ER) [35], we do not expect this to occur in our mitochondrial in situ preparations because of lack of ER Ca^2+^ accumulation in the absence of ATP, which is diluted in the medium.

MICU1-deficient mitochondria of mammalian cells were found to be more sensitive to RuRed or Ru360 inhibition of Ca^2+^ uptake than control mitochondria [29]. This was attributed to the presence of a DIME-interacting domain (DID) in MICU1, comprising two arginines (R440 and R443) and responsible for the interaction of MICU1 and the exposed D-ring of MCU oligomer. This interaction is required for keeping the MCU complex pore closed at low cytosolic Ca^2+^ concentrations ([Ca^2+^]_c_) and optimally activated at high [Ca^2+^]_c_ [29]. This interaction also controls the complex accessibility for its inhibitors, which would also interact with the D-ring of the MCU oligomers [26,27]. Interestingly, the two arginines of the DID are conserved among the three mammalian MICUs [29] as well as in TcMICU1 and TcMICU2 [20]. However, only mitochondria of *MICU1*-KO mammalian cells were tested [29]. In this work, we show that mitochondria of both *TcMICU1*-KO and *TcMICU2*-KO cells are more sensitive to Ru360 and Ru265 inhibition. The results suggest that both TcMICU1 and TcMICU2 would interact with the D-ring of the TcMCU oligomer and that in the absence of any of them, the complex becomes more accessible and more sensitive to inhibition by ruthenium-derivatives. In mammalian cells, ablation of MICU1 leads to decreased protein levels of MICU2 [14]. It is possible that this does not occur in trypanosomes, and upon ablation of one of them, the other subunit is still able to act as a gatekeeper of the channel. The interaction of both proteins with the D-ring would explain why individual ablation of each TcMICU protein does not increase Ca^2+^ uptake at low [Ca^2+^]_c_ [20], as it occurs in mammalian cells [23], as the other subunit will still be present. In mammalian cells, the threshold for Ca^2+^ uptake is affected by the loss of either MICU1 or MICU2, as shown by increased uptake rate when given [Ca^2+^]_c_ below the threshold [23].

It is possible that the different sensitivity of mitochondria of trypanosomes to ruthenium derivatives is due to the different composition of the TcMCU complex. The pore of the TcMCU complex is formed by the contribution of different monomers (MCU, MCUb, MCUc, and MCUd) in contrast to the homo-oligomeric nature of the mammalian uniporter. Finally, experiments with intact cells suggest that ruthenium derivatives have low permeability in trypanosomes. It is interesting to note that prolonged incubation times with high concentrations of Ru265 were needed to see effects on respiration in mouse cortical neurons [36]. The reason for the different permeability to Ru265 could be related to the differences in membrane composition. *T. cruzi* plasma membrane is rich in ergosterol and GPI-anchored proteins [37], and it has been reported that the sterol composition affects its sensitivity to digitonin permeabilization [38]. New derivatives with increased permeability will be needed to study the function of the TcMCU complex in vivo.

## 4. Materials and Methods

### 4.1. Chemicals and Reagents

Calcium green-5N was from Thermo Fisher Scientific (Waltham, MA, USA). Blasticidin S HCl was purchased from Life Technologies (Grand Island, NY, USA). Ruthenium red, ruthenium 360, carboxyatractyloside (CAT), oligomycin, safranine O, antimycin A, carbonylcyanide *p*-trifluoromethoxyphenylhydrazone (FCCP), oligomycin A, G418, and all other reagents of analytical grade were from Sigma (St. Louis, MO, USA). Ru265 was a gift from Justin J. Wilson and Muniswamy Madesh (Department of Medicine, University of Texas Health San Antonio, San Antonio, TX, USA).

### 4.2. Culture Methods

*T. cruzi* epimastigotes (Y strain) were grown in liver infusion tryptose (LIT) medium (5.4 mM KCl, 150 mM NaCl, 24 mM glucose, 5% [vol/vol] liver extract, 0.02% [wt/vol] hemin, 2% [wt/vol] yeast extract, 1.5% [wt/vol] tryptose) [39] containing 10% heat-inactivated newborn calf serum at 28 °C. Mutant cell lines were maintained in medium containing 250 μg/mL G418 and 10 μg/mL blasticidin. The growth rate of epimastigotes was determined by counting cells in a Beckman Coulter analyzer. Tissue culture cell-derived trypomastigotes were obtained from Vero cells infected with metacyclic trypomastigotes. *T. cruzi* trypomastigote forms were collected from the culture medium of infected host cells, using a modification of the method of Schmatz and Murray as described previously [40]. Vero cells were grown in RPMI supplemented with 10% fetal bovine serum and maintained at 37 °C with 5% CO_2_.

### 4.3. Ca^2+^ Uptake by Digitonin-Permeabilized T. cruzi Epimastigotes

Cells were collected by centrifugation at 1000× *g* for 7 min and washed twice with buffer A with glucose (BAG: 116 mM NaCl, 5.4 mM KCl, 0.8 mM MgSO_4_, 5.5 mM d-glucose, and 50 mM HEPES, pH 7.0). Epimastigotes were resuspended to a final density of 1 × 10^9^ cells/mL in BAG and kept on ice. Before each experiment, a 50 μL aliquot of *T. cruzi* epimastigotes (5 × 10^7^ cells) was added to the reaction buffer (125 mM sucrose, 65 mM KCl, 10 mM HEPES-KOH buffer, pH 7.2, 1 mM MgCl_2_, 2.5 mM potassium phosphate [1.95 mL]) containing 5 mM succinate, 50 μM EGTA, and 0.5 μM fluorescent cell-impermeable Ca^2+^ indicator calcium green-5N. Mitochondrial Ca^2+^ uptake was initiated by the addition of ~30 μM of free calcium, which was calculated using the software Maxchelator Calculator v1.2 (https://somapp.ucdmc.ucdavis.edu/pharmacology/bers/maxchelator/CaEGTA-NIST.htm). This calcium addition was followed by 50 μM digitonin and 4 μM FCCP. Fluorescence changes were monitored in an F-7000 fluorescence spectrophotometer (Hitachi, Dallas, TX, USA) with excitation at 506 nm and emission at 532 nm. The relative rates of Ca^2+^ uptake were normalized for the control strains. The hyperbolic equation [Ca^2+^] = *K_d_* × [(*F* − *F*_min_)/(*F*_max_ − *F*)] was used to convert the raw fluorescence readings measured during mitochondrial Ca^2+^ transport assays into Ca^2+^ concentration levels, where *K_d_* is the dissociation constant, *F* is any given fluorescence value, *F*_min_ is the lowest fluorescence reading after addition of 0.5 mM EGTA, and *F*_max_ is the maximal fluorescence obtained after two sequential additions of 1 mM CaCl_2_. These additions were performed at the end of each trace. *K_d_* for Ca^2+^ indicator probes in our conditions was determined, according to Chweih et al. [41]. Uptake rate inhibitions were calculated as the first derivative of the absolute values of the slope by using the SLOPE Excel function for 200 points (300 to 500 s). Slope values were transformed into logarithm values and normalized as percentage values. Dose-response curves were compiled, fitting the normalized values in a sigmoidal curve.

### 4.4. Mitochondrial Membrane Potential

Estimation of mitochondrial membrane potential in situ was done spectrofluorometrically using the indicator dye safranine O, as described previously [19]. Briefly, *T. cruzi* epimastigotes (5 × 10^7^ cells) were incubated at 28 °C in reaction buffer (125 mM sucrose, 65 mM KCl, 10 mM HEPES-KOH buffer, pH 7.2, 1 mM MgCl_2_, 2.5 mM potassium phosphate [1.95 mL]) containing 5 mM succinate, 0.2% BSA, 50 μM EGTA, and 5 μM safranine O, and the reaction was started with digitonin (50 μM). ADP (250 μM), carboxyatractyloside (1.5 μM), and FCCP (4 μM) were added to the medium at different time points. Fluorescence changes were monitored using the Hitachi F-7000 spectrofluorometer (excitation of 495 nm and emission of 586 nm).

### 4.5. Cellular Respiration

Cells were collected by centrifugation at 1000× *g* for 7 min and washed twice with buffer A with glucose (BAG: 116 mM NaCl, 5.4 mM KCl, 0.8 mM MgSO_4_, 5.5 mM d-glucose, and 50 mM HEPES, pH 7.0). Epimastigotes were resuspended to a final density of 1 × 10^9^ cells/mL in BAG and kept on ice. Cells (1 × 10^8^) were incubated at 28 °C in a 2 mL chamber containing buffer A with glucose (with or without 10 μM Ru265). Oligomycin (5 μg/mL) and FCCP (0.2 μM) were sequentially added. The oxygen consumption rates of epimastigotes were measured using a high-resolution respirometer (Oroboros Oxygraph-2k; Oroboros Instruments GmbH, Innsbruck, Austria). The equipment was calibrated as reported by its manufacturer. OCR was calculated as the negative time derivative of the oxygen concentration measured in the close respirometer chambers and expressed per milligram of protein.

### 4.6. Measurement of Mitochondrial Ca^2+^ Uptake in Intact Epimastigotes Using Rhod-2 AM

Cells were collected by centrifugation at 1000× *g* for 7 min and washed twice with buffer A with glucose (BAG: 116 mM NaCl, 5.4 mM KCl, 0.8 mM MgSO_4_, 5.5 mM d-glucose, and 50 mM HEPES at pH 7.0). Epimastigotes were resuspended to a final density of 1 × 10^9^ cells/mL in BAG and loaded with Rhod-2 AM (2 µM; 50 min). The cells were washed twice with buffer A with glucose and pretreated with or without 50 μM Ru265 for 30 min. Before each experiment, a 50 μL aliquot of epimastigotes (5 × 10^7^ cells) was added to the BAG. Mitochondrial Ca^2+^ uptake occurred after the addition of 1 μM nigericin (Nig) and 1 μM ionomycin (Ion) to rapidly release Ca^2+^ from neutral and acidic compartments. The addition of 8 μM FCCP increased the release of mitochondrial Ca^2+^. Fluorescence changes were monitored in an F-7000 fluorescence spectrophotometer (Hitachi) with excitation at 552 nm and emission at 581 nm.

### 4.7. Attachment Assay

Gamma-irradiated (2000 rads) Vero cells were grown at 37 °C in 24-well plates on a 13 mm-diameter round glass coverslip at a density of 2 × 10^5^ cells/well in 7% CO_2_ in RPMI medium plus 10% fresh fetal bovine serum. After 24 h, the medium was removed, and the cells were washed twice with Hanks solution and prefixed for 5 min at 4 °C with 2% glutaraldehyde in phosphate-buffered saline (PBS). After fixation, the cells were immediately washed with PBS. Prior to incubation with the cells, the parasites were preincubated with 1 μM antimycin A or 10 μM Ru265. After 30 min of incubation at 37 °C, the parasites were seeded onto Vero cells (5 × 10^6^ parasites/well). After 1 h of incubation at 37 °C, the coverslips were washed once with PBS and mounted onto glass slides in Fluoromount-G containing 15 μg/mL of 2-(4-aminophenyl)-1H-indole-6-carboxamidine (DAPI), which stains host and parasite DNA. Coverslips were viewed on an Olympus BX60 microscope to quantify the number of host cells that contained adherent parasites and the number of adherent parasites per cell in randomly selected fields. Three hundred host cells were counted per sample in four independent experiments.

### 4.8. In Vitro Infection Assay

Gamma-irradiated (2000 rads) Vero cells (4.5 × 10^5^ cells) were plated onto sterile coverslips in a 12-well plate and incubated overnight at 35 °C in 7% CO_2_ in RPMI medium plus 10% fresh fetal bovine serum. Tissue culture-derived trypomastigote collections were incubated at 4 °C overnight to allow amastigotes to settle from swimming trypomastigotes. Trypomastigotes from the supernatants of these collections were counted and used to infect the coverslips at a ratio of 50 parasites to 1 host cell. Prior to incubation with the cells, the parasites were preincubated with 1 μM antimycin A or 10 μM Ru265 for 30 min. At 4 h post-infection, coverslips were washed extensively with Hanks’ solution, followed by phosphate-buffered saline (PBS) at pH 7.4 to remove any extracellular parasites. Coverslips were fixed immediately in 4% paraformaldehyde in PBS (pH 7.4) at 4 °C for 30 min. Coverslips were washed once with PBS and mounted onto glass slides in Fluoromount-G containing 15 μg/mL of 2-(4-aminophenyl)-1H-indole-6-carboxamidine (DAPI), which stains host and parasite DNA. Coverslips were viewed on an Olympus BX60 microscope to quantify the number of host cells that contained intracellular parasites and the number of intracellular parasites per cell in randomly selected fields. Three hundred host cells were counted per sample in four independent experiments.

### 4.9. Statistical Analysis

Statistical analyses were performed with GraphPad Prism software version 8.4 (GraphPad, La Jolla, CA, USA). Reported values are means ± standard deviation (SD) from *n* biological experiments, as indicated in figure legends. The level of significance was evaluated by one-way analysis of variance (ANOVA) for comparisons between more than two cell lines and two-way ANOVA with multiple-comparison tests for analyses of grouped data.

## Abbreviations

MCU	Mitochondrial calcium uniporter
MICU	Mitochondrial calcium uptake
MCUR1	MCU regulator 1
RuRed	Ruthenium red
